# Design and Evaluation of NSAID Derivatives as AKR1C3 Inhibitors for Breast Cancer Treatment through Computer-Aided Drug Design and In Vitro Analysis

**DOI:** 10.3390/molecules29081802

**Published:** 2024-04-16

**Authors:** Victoria Fonseca-Benítez, Paola Acosta-Guzmán, Juan Esteban Sánchez, Zaira Alarcón, Ronald Andrés Jiménez, James Guevara-Pulido

**Affiliations:** Investigación en Química Aplicada INQA, Química Farmacéutica, Universidad El Bosque, Bogotá 11001, Colombia; avfonseca@unbosque.edu.co (V.F.-B.); zalarcon@unbosque.edu.co (Z.A.);

**Keywords:** CADD, artificial neural networks (ANNs), organic synthesis, breast cancer

## Abstract

Breast cancer is a major global health issue, causing high incidence and mortality rates as well as psychological stress for patients. Chemotherapy resistance is a common challenge, and the Aldo-keto reductase family one-member C3 enzyme is associated with resistance to anthracyclines like doxorubicin. Recent studies have identified celecoxib as a potential treatment for breast cancer. Virtual screening was conducted using a quantitative structure–activity relationship model to develop similar drugs; this involved backpropagation of artificial neural networks and structure-based virtual screening. The screening revealed that the C-6 molecule had a higher affinity for the enzyme (−11.4 kcal/mol), a lower half-maximal inhibitory concentration value (1.7 µM), and a safer toxicological profile than celecoxib. The compound C-6 was synthesized with an 82% yield, and its biological activity was evaluated. The results showed that C-6 had a more substantial cytotoxic effect on MCF-7 cells (62%) compared to DOX (63%) and celecoxib (79.5%). Additionally, C-6 had a less harmful impact on healthy L929 cells than DOX and celecoxib. These findings suggest that C-6 has promising potential as a breast cancer treatment.

## 1. Introduction

Breast cancer (BC) is the third most common type of cancer worldwide and the second leading cause of cancer deaths among women [1]. BS presents biological diversity and high heterogeneity due to specific hormone receptor expression, mainly responding to therapy. Owing to its heterogeneous nature, chemoresistance is one of the challenges in BC, and its mechanisms have not been fully elucidated [2]. Several studies have demonstrated that the AKR1C3 enzyme is related to some steroid hormones due to regulation, such as AR (androgen), ER (estrogen), and PgR (progesterone) [3]; it is overexpressed in several hormone-related cancers and is correlated with tumor development and resistance to anthracyclines such as doxorubicin, one of the first choices in breast cancer treatment [4]. Consequently, developing highly potent and specific AKR1C3 inhibitors to restore the chemosensitivity of drug-resistant breast cancer has become an essential research topic. Previous reviews showed that in preclinical and clinical studies, celecoxib demonstrated promising results in treating and preventing cancer. The best outcome was observed in colon, breast, prostate, and head and neck cancers [5]. Previous works showed that celecoxib can inhibit AKR1C3 activity at physiological concentrations [6]. These results prove that celecoxib could be a promising adjuvant chemotherapy drug to improve breast cancer treatment outcomes [7,8].

Considering that celecoxib has a good affinity for the AKR1C3 enzyme, its activity has been verified in clinical studies against BC [9]. In this study, we aim to enhance a drug’s pharmacokinetic and dynamic properties by making systematic changes to the auxophoric scaffold. Metabolic interferences often occur in the auxophoric groups [10]. Hence, we attempted modifying the phenyl sulfonamide in celecoxib through isosteric or homologous modifications. These structural modifications offer superior synthetic benefits, as they are more reactive functional groups than aromatic rings. It enables high chemical yields without any chemo- and regioselectivity issues. Considering the importance of having more selective molecules for molecular targets, we used the rational design of drugs as a strategy that allows better drugs to be obtained faster at lower costs, with the attempt to reduce the effects associated with low selectivity as much as possible [11,12,13,14,15,16,17].

Virtual screening (VS) is divided into structure-based virtual screening (SBVS) that uses organic molecules in their 3D form to assess their affinity against molecular targets using tools such as molecular docking in auto dock vina software [18,19]. Similarly, ligand-based virtual screening (LBVS) allows pharmacological parameters to be predicted, such as values of half-maximal inhibitory concentration (IC_50_) and the logarithm of the partition coefficient (log P) cytotoxic concentration, through quantitative structure–activity relationship (QSAR) models and their structure–activity relationships (SAR) [20]. Partial least squares and multiple linear regression are some strategies for building predictive models. However, one of the most popular is artificial neural networks (ANNs) because it is possible to make predictions with good coefficients of determination in models that do not necessarily have linear relationships [15]. Our research group built the architecture of an ANN that was used for the development of QSAR models and, with the molecular docking analysis, allowed us to design, synthesize, and evaluate in vitro new and safer SSRIs [21], SARS-CoV-2 inhibitors [22], and new organic UV filters [23]. We created these by LBVS, and SBVS synthesized the top candidate. We evaluated the biological activity of a celecoxib analog, with a higher affinity for AKRIC3, lower IC_50_ values, and safer toxicity profile, as a possible candidate for the treatment of breast cancer.

## 2. Results and Discussion

### 2.1. Bioinformatic

DrugBank, ChEMBL, and Inxight Drugs databases were initially reviewed to identify molecules with experimental inhibition values on the AKR1C3 aldo-keto reductase family member C3 [24]. After the screening, 40 commercial molecules such as NSAIDs, alkaloids, benzodiazepines, cyclopentenones, flavonoids, and steroids were included (Table S1) [25]. However, we performed a second screening, looking for the steric and electronic groupings of the compounds to have equivalent pharmacophoric groups, reducing the list to 12 NSAIDs. Two NSAIDs were dismissed after being identified as outliers with a boxplot (Figure S1). In parallel, the QSAR model based on ANNs was built in MATLAB with the previously described architecture [22,26,27], with the experimental values of IC_50_ of the commercial NSAIDs as the training set (Table 1, entries 1–10).

Subsequently, a systematic screening of the molecular descriptors, which were previously calculated with PaDEL [28], was conducted, and the following descriptors were selected employing Pearson correlations (Figure S2): naAromAtom, TopoPSA, and McGowan_Volume, (Table 2). We began to build the model with the filtered molecular descriptors, and tests from 50 to 1200 hidden nodes yielded a predictive model with a determination coefficient (R^2^) of 0.679. This model was then used to calculate the theoretical IC_50_ values of the training set to validate the model (Figure S3). With the experimental IC_50_ values, celecoxib was selected as the molecular framework. The analogs were designed because they exhibited more affinity by AKR1C3 (Table 1), and more studies have demonstrated its value in breast cancer [4,25]. The molecular descriptor with a built model and the designed analogs’ molecular descriptors were calculated, and their IC_50_ value was calculated. (Table 1, entries 11–30). As previously described, in parallel, the study employing SBVS was carried out to evaluate the binding affinity of the commercial NSAIDs and the designed analogs for the active site AKR1C3. Table 1, entries 1–30, illustrate the average binding affinity for each compound. Selection of the best AKR1C3 inhibitors was based on four criteria: (i) structures with lower predicted IC_50_ values than the one for celecoxib (2.3 µM), (ii) structures that showed more negative binding affinity to the active site compared to celecoxib (−10.4 kcal/mol) (Table 1, entry 7), (iii) structures with toxicity profiles similar or more favorable than celecoxib as calculated by LAZAR toxicity predictions (Table 3) [29], and (iv) structures from 2.5 to 5.0 LogP. (Table 1, entries 1–30).

**Scheme 1:**
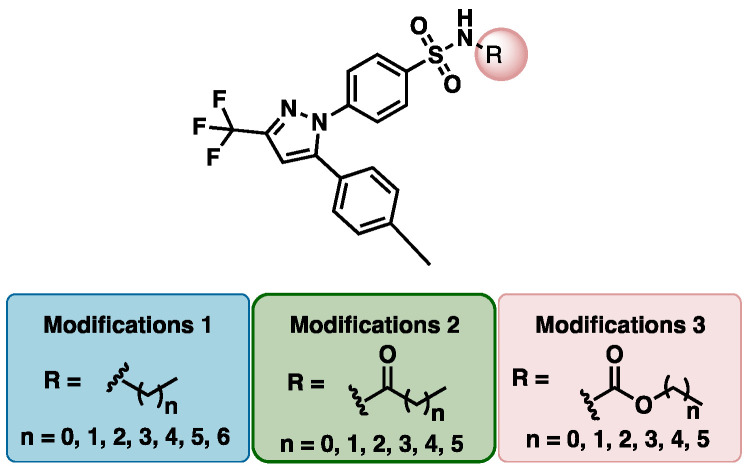
Rationales modifications of phenyl sulfonamide of celecoxib. Modifications 1 (A1–A6), Modifications 2 (B1–B6), and Modifications 3 (C2–C7).

Consequently, for the rational design of the analogs, we used homologous series to modify the structure of celecoxib with *N*-alkylbenzenesulfonamides substitutions (Table 1, entries 11–16), alkyl (phenylsulfonyl)carbamates (Table 1, entries 17–24), and *N*-(phenylsulfonyl) alkylamides substitutions (Table 1, entries 25–30) (Scheme 1).

Compared to commercial celecoxib, 10 of the 20 proposed structures had affinity values (SBVS) that were equally or even more harmful for AKR1C3. The preferred interactions between AKR1C3 and 3-(3,4-dihydroisoquinolin-2(1*H*)-ylsulfonyl)benzoic acids were determined to be Tyr216, Phe311, Tyr219, and Hys119 in previous studies [30,31]. Interactions were preserved, and analogs C-6 and C-7 enhanced the number of hydrophobic interactions compared to our developed counterparts, which may cause an increase in interaction energy (SI). Keeping with LVBS, six analogs of all those created had lower IC_50_ predicted by ANN compared to celecoxib (Table 1, entries 25–30). The fact that celecoxib’s whole pharmacophoric grouping was kept and the auxophoric chain of the sulfonamide was explicitly altered, which has little to no impact on the calculation of the descriptors between the analogs, accounts for the similarity of the anticipated IC_50_ values in most created analogs. As a result, six analogs belonging to the *N*-(phenylsulfonyl) alkylamides were produced (modification 2, Scheme 1). We selected this group’s lowest IC_50_ (Table 1, entries 29–30) values. The toxicity of a substance was predicted by analyzing two rodent carcinogenicity studies and a mutagenicity assay using *S. typhimurium* (Table 3). PreADMET utilized a model developed with information from the National Toxicology Program and the U.S. Food and Drug Administration to anticipate the results of two-year in vivo carcinogenicity studies on mice and rats. One of the essential factors in drug development is the n-octanol/water partition coefficient (log Po/w). To estimate this value, the SwissADMET program uses a reliable GB/SA approximation based on more than 17,000 chemicals (Table 1, entries 29–30) [32]. This kept the C-6 comparable, which satisfied the four initial requirements.

The improvement in the pharmacokinetic and dynamic features of the celecoxib analogs can be attributed to three key reasons, as shown by the in silico studies. Firstly, the affinity values for AKR1C3 are increased by adding a hydrogen bond-acceptor group, such as carbamate or alkylamides. Secondly, as predicted, the LogP value rises with the increase in methylenes. Alkylamide combinations with five carbons can reduce the IC_50_ value, illustrating the versatility of combining ligand-based virtual screening with virtual structure-based screening as a tool for the rational design of drugs. However, adding carbons should not exceed five to alkylamines and four to carbamates because it exceeds the allowed reference values. 

A radar plot tool was used to analyze the molecular structures of celecoxib and its C-6 analog and compare their drug-likeness properties. This analysis helped us understand the similarities and differences between the two compounds.

**Figure 1:**
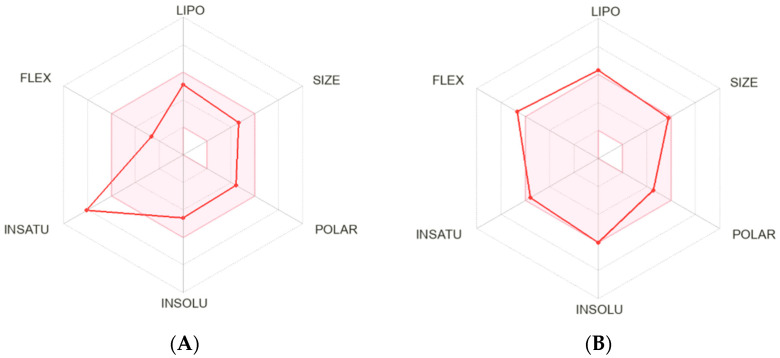
Radar plot celecoxib (**A**) and C-6 analog (**B**).

According to the SwissADME calculation, the pink area indicates a good property space for oral bioavailability. At the same time, the red hexagon represents the values of six calculated properties for celecoxib (Figure 1A) and C-6 (Figure 1B). Candidate C-6 yielded better properties, so we proceeded with the chemical synthesis process.

### 2.2. Synthesis and Characterization

**Scheme 2:**
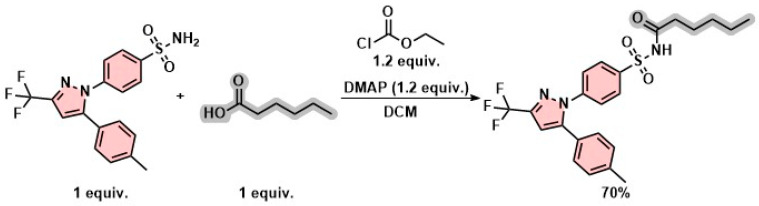
Synthesis of C-6 *N*-((4-(5-(*p*-tolyl)-3-(trifluoromethyl)-1*H*-pyrazol-1 yl)phenyl) sulfonyl) hexanamide.

In a 25 mL flask, hexanoic acid (26 μL, 0.26 mmol, 1 eq.), DMAP (0.31 mmol, 1.2 eq.), and ethyl chloroformate (29 μL, 0.31 mmol, 1.2 eq.) in DCM (3 mL) were stirred for 30 min. Then, celecoxib was added in three portions (100 mg, 0.26 mmol, 1.0 eq.). The mixture was stirred at room temperature for approximately 12 h. The progress of the reaction was monitored using thin-layer chromatography. Once complete consumption of the starting material was observed (formation of a new product with a similar Rf value as celecoxib), water was added, and extractions were performed with DCM (3 × 20 mL). The organic phases were collected, dried over anhydrous Na_2_SO_4_, and filtered, and the solvent was removed under reduced pressure. The crude reaction mixture was purified by flash column chromatography using cyclohexane: CH_2_Cl_2_ (1:1) mobile phase. The product was obtained as a white solid (86.3 mg, 0.18 mmol, 70%), m.p. 300 °C, as shown in Scheme 2. C-6 was characterized as *N*-((4-(5-(*p*-tolyl)-3-(trifluoromethyl)-1*H*-pyrazole-1 yl)phenyl) sulfonyl) hexanamide (Scheme 2). ^1^H NMR (400 MHz, Methanol-d_4_) δ 7.93 (d, J = 8.7 Hz, 2H), 7.48 (d, J = 8.7 Hz, 2H), 7.09–7.30 (m, 4H), 6.90 (s, 1H), 2.34 (s, 3H), 2.27 (t, J = 7.5 Hz, 2H), 1.49–1.68 (m, 2H), 1.16–1.42 (m, 4H), 0.91 (t, J = 6.9 Hz, 3H). ^13^C NMR (101 MHz, MeOD) δ 177.7, 147.0, 145.0, 144.8 (q, J = 38 Hz) 143.2, 141.0, 130.6, 130.0, 128.3, 127.2, 127.0, 122.7 (q, J = 268.7 Hz),106.9, 48.6, 34.9, 32.4, 25.8, 23.4, 21.3, 14.3. FT-IR (neat) u(cm^−1^): 3334, 3228, 2928, 1708, 1345, 1133, 1101. HRMS (ESI): C_23_H_25_F_3_N_3_O_3_S^+^ [M + H^+^]: calc. 480.1563, found. 480.1566.

### 2.3. Viability Assay

**Figure 2:**
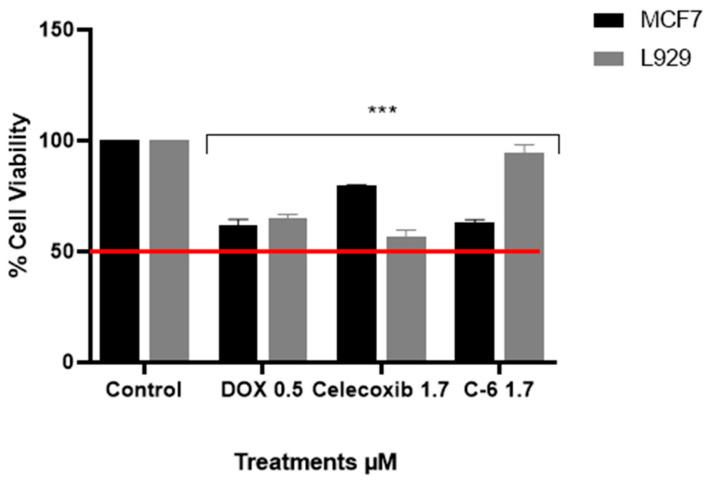
Cell viability profile of MCF7 and L929 cells treated with doxorubicin, celecoxib, and C-6. The cells were treated with concentrations of the drugs shown in Table 1. Each bar represents the median and interquartile range of four independent experiments and a 24 h evaluation of untreated control (*** *p* < 0.005).

To test C-6 and celecoxib’s cytotoxic effects after 24 h of treatment, we used MCF7 cells. These cells are commonly used to study chemotherapy resistance in breast cancer and express the AKR1C3 receptor, associated with hormone-dependent breast cancer [33]. The drug concentrations obtained in silico analysis, as shown in (Table 1, entry 29), were used. The results received after 24 h on MCF7 cells treated with celecoxib showed a cell viability of 79.5%, while doxorubicin and C-6 showed similar behavior with percentages of 62% and 63%, respectively. Regarding safety in L929, both doxorubicin and celecoxib showed high toxicity and low selectivity on healthy cells, with viability results of 64% and 57%, respectively. However, C-6 showed excellent safety, with viability percentages more significant than 90% (Figure 2).

**Figure 3:**
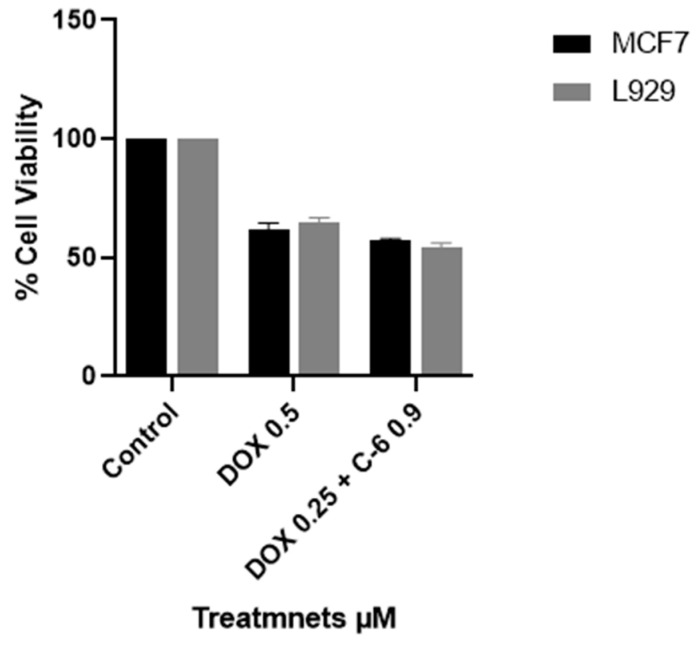
Cell viability profile of effects on MCF7 and L929 cells treated with doxorubicin and C-6 as adjuvant therapy. The cells were treated with concentrations of doxorubicin 0.25 µM and C-6 0.9 µM. Each bar represents the median and interquartile range of four independent experiments for a 24 h evaluation concerning the control (*p* < 0.005).

Finally, considering that AKR1C3 is widely related to resistance to doxorubicin [34], we wanted to evaluate a possible synergistic effect between DOX and C-6 at half the IC_50_ concentrations previously assessed. However, the results showed a high cytotoxic effect on tumors and healthy cells, and this reveals that C-6 is a potential therapeutic candidate for hormone-dependent and anthracycline-resistant breast cancer since it showed high selectivity and low toxicity (Figure 3).

In the first measure, our results prove the effectiveness of the in silico analysis. It was determined that the affinity in kcal/mol for AKR1C3 improves when phenylsulfonamide is replaced by *N*-(phenylsulfonyl) alkylamines, and the IC_50_ value decreases as methyl groups are added to the chain. However, when the homologous series of cargo bonds increases to seven, the LogP value goes out of the range, which limits its application. Therefore, the rise in lipophilicity and the addition of a hydrogen bond acceptor group improves the pharmacokinetics and dynamics of the designed candidates. The data obtained in the network were verified in the cell viability assay. In addition, obtaining a more selective molecule for tumor cells with excellent safety was possible. However, more biological studies are required to evaluate the mechanisms of cell death triggered by the inhibition of AKR1C3 and the evaluation of the inhibitory effect on the tumor cell molecular behavior.

## 3. Materials and Methods

### 3.1. Bioinformatics

#### 3.1.1. Ligand-Based Virtual Screening (LBVS)

We built a QSAR model using the architecture of ANNs, which was previously described and validated. Molecules filtered from the literature were drawn in Avogadro software, and their energy was minimized to 0.0 with the MMFF94s force field [35]. After placing the molecules in .mol format, 1370 molecular descriptors were calculated using the PaDEL software. Next, a screening of the descriptors calculated using MATLAB was performed under the following criteria: (i) elimination of atypical data, (ii) elimination of zeros, (iii) descriptor vs. descriptor correlation between 0 and 0.4, and (iv) descriptor vs. IC_50_ correlation close to 1.0. The descriptors that exceeded the mentioned criteria are assigned as input data, and the variable to be predicted is the IC_50_. The validation model was carried out by cross-validation by the leave-one-out method, and the validation criterion was determined by a value greater than 0.6 of its correlation coefficients (R^2^), increasing the nodes of the hidden layer up to 1200.

#### 3.1.2. Structure-Based Virtual Screening (SBVS)

Each molecule affinity was determined to the AKR1C3 crystal registered in PDB 3R58 that met the quality parameters. The enzyme was conditioned according to the software protocol [36]. Molecules filtered from the literature were constructed in Avogadro software, and their energy was minimized to 0.0 with the MMFF94s force field. Then, ten commercial nonsteroidal anti-inflammatory (NSAID) inhibitors were docked with the binding site of AKR1C3. The grid box size and coordinates were adjusted, respectively, to 26 × 28 × 26 and −0.361, −2.778, and −9.722. Calculations were performed in triplicate, and the affinity energy (kcal/mol) of the pose with the lowest RMSD value was averaged for each molecule. The same protocol was used for the celecoxib analogs that were designed. Finally, the interactions and distances were visualized in Discovery Studio Suite^®^.

#### 3.1.3. Toxicity and LogP Profile

The toxicity properties and logP value of celecoxib and the designed analogs were predicted in silico using the PreADMET 2.0 SwissADME 1.0 software. Molecules with the same or better toxicity profile than the commercial drug and those with a logP value between 2.5 and 4.9 were selected.

(https://preadmet.webservice.bmdrc.org) PreADMET procedure: We drew the compounds for which we wished to estimate the toxicity, making sure they did not have any structural flaws, and then used the PreADMET toxicity prediction module to compute the toxicity and compare the carcinogenicity values in rats and mice and the mutagenicity of analogs and commercial NSAIDs.

The SwissADME process (http://www.swissadme.ch) involved building commercial NSAIDs and celecoxib analogs. The program was run, and then, the consensus Log Po/w of commercial NSAIDs and analogs was contrasted.

### 3.2. Chemistry

All reagents used in the experiment were obtained from commercial suppliers and used without further purification. To monitor the progress of the reaction, TLC was performed on aluminum plates coated with silica gel F254 indicator, which was visualized either by UV irradiation or by staining with iodine. Flash chromatography used silica gel 60 (230–240 mesh). For NMR analysis, 1H and 13C spectra were recorded in MEOD using a Bruker Avance NEO 400 MHz spectrometer. The chemical shifts for 1H and 13C were indicated in parts per million (ppm, δ), with tetramethylsilane as the internal reference. The splitting patterns for 1H NMR were designated as singlet (s), doublet (d), triplet (t), quartet (q), and multiplet (m). The coupling constants and integration were quoted in Hertz (J). Infrared spectra were recorded using a Bruker Alpha-P ATR FTIR with a diamond crystal. High-resolution mass spectrometry was carried out using an Agilent 5973 (80 eV) spectrometer with electrospray ionization (ESI). All reagents were used without further purification as received from commercial suppliers.

In the round-bottom flask, a solution of hexanoic acid (99%) (26 μL, 0.26 mmol, 1 eq.), DMAP (99%) (0.31 mmol, 1.2 eq.), and ethyl chloroformate (97%) (29 μL, 0.31 mmol, 1.2 eq.) in DCM (3 mL) were stirred for 30 min. Then, celecoxib was added in three portions (100 mg, 0.26 mmol, 1 eq.). The mixture was stirred at room temperature for approximately 12 h (TLC). Then, the solvent was evaporated in vacuo, and flash chromatography purified the crude mixture (cyclohexane: CH_2_Cl_2_) (1:1). The pure product C-6 was obtained with a yield of 82% (0.30 mmol).

#### Solubility and HPLC Method to C-6 and Celecoxib

In separate solutions, 5 mg of C-6 and celecoxib were added to 10 mL of (PBS, 1X pH 7.35). The mixtures were sonicated for 2 h at room temperature. Subsequently, the result mixtures were centrifuged at 4000 rpm for 20 min. Then, the resulting solutions were filtered, and the solids were discarded. The C-6 and celecoxib solutions in PBS had their area under the curve calculated through HPLC-RP. The method was performed with a flow of 1 mL/min, 255 nm, a mobile phase of (50/50) water/ACN on a reprosil-pur introductory C18 150 × 46 mm column, and the concentration of the solutions in PBS was determined by interpolation of calibration curves of C-6 and celecoxib dissolved in MeOH 1.0 µM to 50 µM.

### 3.3. Cell Lines and Culture Conditions

The cell lines MCF7 (ATCC^®^ HTB-22™) adenocarcinoma breast, mammary gland, and healthy cell line L929 were cultured in DMEM (Eagle modified by Dulbecco) supplemented with 10% fetal bovine serum (FBS-Gibco, Fischerscientific, Alcobendas Madrid Spain). The cells were incubated in a humidified atmosphere and 5% CO_2_ at 37 °C. The cells were given fresh culture media thrice weekly and subcultured at confluence after detaching with 0.25% trypsin-EDTA solution.

#### 3.3.1. Treatments

The cells were treated with the best candidate obtained by the previously described model and evaluated at IC_50_ concentration (1.70 µM). Additionally, celecoxib was synthesized in our laboratory and estimated at 1.70 µM. Doxorubicin hydrochloride (0.5 µM) was obtained from Pharmacia and was used as a death-positive control. Cells not treated were used as controls.

#### 3.3.2. Cytotoxicity Screening

The Alamar blue assay (BioSource et al., San Diego, CA, USA) was used to determine the treatment effect in tumor and normal cells. Cells were seeded in 96-well plates at a density of 5 × 104 cells/well, allowing the attachment for 24 h. The treatment was evaluated for 24 h. After treatment time, the culture medium was replaced by 100 μL of resazurin solution (40 μM), the plates were incubated for four h, and the fluorescence of resorufin was measured in a microtiter plate reader (530–590 nm, Tecan, Infinite^®^ 200 PRO). The cell viability was expressed as the percentage of live cells relative to the untreated control (cell viability control/cell viability treatment) × 100. Profile–response doses of the cell viability percentage plotted against concentrations of the treatments were constructed.

#### 3.3.3. Statistical Analysis

Data were expressed as arithmetic mean ± SEM. Data were analyzed by Origin 2022b (OriginLab Corporation, Northampton, MA, USA). GraphPad Prism software was used to do the graphs. Cytocompatibility assay was analyzed by one-way ANOVA. A *p*-value less than 0.05 was considered statistically significant.

## 4. Conclusions

Various computational tools were used to predict NSAID derivatives’ pharmacokinetic and dynamic parameters, including SBVS, LBVS, LAZAR toxicity, and SwissADME. These tools helped us determine the affinity in kcal/mol for AKR1C3, IC_50_, toxicity, and LogP value. Analog C-6 exhibited a binding affinity of −11.4 kcal/mol and a predicted IC_50_ value of 1.7 µM, which was better than celecoxib. We then synthesized and characterized the analog and evaluated its cytotoxicity using a tumor cell line (MCF7) and healthy cell line (L929). Our results showed that C-6 had similar behavior to DOX, with 62% and 63% cell viability percentages, respectively. Furthermore, it performed better than celecoxib, with a cell viability percentage of 79%. Notably, C-6 was deemed safe for use as a candidate for treating breast cancer, as it exhibited more significant cell viability percentages than 90% in the healthy cell line (L929). This makes it a promising candidate for future evaluations, including in vivo assays.

## Figures and Tables

**Table 1:** LBVS and SBVS results for inhibitors of AKR1C3 and the designed celecoxib analogs.

Entry	Compound	LBVS Exp IC_50_ (µM)	Pred IC_50_ ^a^ (µM)	SBVS Affinity ^b^ (kcal/mol)	Lipophilicity Pred ^c^
1	Naproxen	0.5	0.6	−8.6	2.76
2	Diclofenac	2.6	1.2	−8.9	3.66
3	Flurbiprofen	7.8	4	−9.3	3.59
4	Lornoxicam	0.7	0.6	−8.7	1.50
5	Mefenamic acid	0.3	0.1	−9.0	3.30
6	Ibuprofen	33.0	30	−7.7	3.00
7	Celecoxib	5.2	2.3	−10.4	3.40 ^d^
8	Ketoprofen	6.0	3.0	−9.0	2.84
9	Sulindac	3.4	3.6	−9.9	3.96
10	Indomethacin	2.3	0.4	−9.4	3.63
11	A1	--	2.0	−9.4	3.75
12	A2	--	2.2	−9.3	4.03
13	A3	--	2.3	−9.5	4.35
14	A4	--	2.2	−9.6	4.74
15	A5	--	2.0	−9.3	5.10
16	A6	--	2.1	−9.4	5.42
17	B1	--	2.2	−10.7	3.50
18	B2	--	2.3	−10.9	3.82
19	B3	--	2.3	−11.1	4.22
22	B4	--	2.5	−11.5	4.55
23	B5	--	2.4	−10.1	4.86
24	B6	--	2.4	−10.1	5.14
25	C2	--	2.1	−11.4	3.46
26	C3	--	1.9	−11.3	3.76
27	C4	--	1.9	−11.2	4.10
28	C5	--	1.8	−11.1	4.46
29	C6	--	1.7	−11.4	4.81 ^d^
30	C7	--	1.7	−11.0	5.16

Entry 1–10: training set; entry 11–25: test set: Exp., experimental; pred., predicted. ^a^ Predicted IC_50_ with a QSAR model. ^b^ Average binding affinity for AKR1C3 (PDB 3R58). ^c^ Consensus Log *P* o/w predicted by swissADMET. ^d^ Solubility in PBS determined by HPLC-RP method (SI) (celecoxib 4.3 µM and C-6 5.1 µM).

**Table 2:** Selected descriptors.

Descriptor	Description
*naAromAtom*	Number of aromatic atoms
*TopoPSA*	Topological polar surface area
*McGowan_Volume*	Volume of a mole when the molecules are not in motion

**Table 3:** In silico toxicity evaluation of celecoxib and selected analogs.

Compound	Mutagenicity	Carcinogenicity in Rats	Carcinogenicity in Mice
Celecoxib	Non-mutagen	Negative	Positive
C-6	Non-mutagen	Negative	Negative
C-7	Non-mutagen	Negative	Negative

## Data Availability

Additional required data will be shared by writing to the corresponding author’s email.

## Supplementary Materials

The following supporting information can be downloaded at https://www.mdpi.com/article/10.3390/molecules29081802/s1.
