# Supplementary material for: Design and Evaluation of NSAID Derivatives as AKR1C3 Inhibitors for Breast Cancer Treatment through Computer-Aided Drug Design and In Vitro Analysis
Source: Molecules. 2024 Apr 16;29(8):1802. doi: 10.3390/molecules29081802 (PMC11052204; doi:10.3390/molecules29081802)

Design and Evaluation of NSAID Derivatives as AKR1C3 Inhibitors for Breast Cancer Treatment through Computer-Aided Drug Design and In Vitro Analysis

Victoria Fonseca-Benítez,[a] Paola Acosta-Guzmán,[a] Juan Esteban Sanchéz,[a] Zaira Alarcó n,[a] Ronald Andrés Jímenez,[a] James Guevara-Pulido* [a]

INQA , Química Farmacéutica, Facultad de Ciencias,[b] Universidad El Bosque [b]

*joguevara@unbosque.edu.co

Table S1 2

BIOINFORMATICS 3

Figure S1 Boxplot outliers 3

Figure S2 Pearson correlations descriptor vs descriptor 4

Figure S3 Pearson correlations descriptor vs IC50 4

Figure S4 Goodness of fit and cross-validation of the QSAR mode 5

INTERACTIONS 5

CHEMISTRY 6

NMR spectra 6

IR-ATR Spectra 8

HPLC C-6 8

Table S1

MOLECULES	(Kcal/mol)	IC_{50} (µM)
Naproxen	-8,6	0,48
Diclofenac	-8,9	2,6
Flurbiprofen	-9,3	7,8
Lornoxicam	-8,7	0,7
Mefenamic acid	-9,0	0,3
Ibuprofen	-7,7	33
Celecoxib	-10,4	5,2
Ketoprofen	-9,0	6
Sulindaco	-9,9	3,4
Indomethacin	-9,4	2,3
Salicylic acid	-10,4	770
Acetylsalicylic acid	-9,8	1200
Stylopine	-11,5	7,7
Canada	-10,2	29
Diazepam	9,8	84
Medazepam	8,8	116
Estazolam	9,8	47
Flunitrazepam	10,3	58
Cloxazolam	9,4	2,5
Bromazepam	9,3	8100
Oxazolam	6,5	2100
Oxazepam	10,3	1400
jasmonic acid	-7,2	21
A1	-8,1	36,7
A2	-7,9	17,8

A3	-8,0	22,5
A4	-8,5	2,7
A5	-9,8	67,3
A6	-10,1	46,6
A7	-8,0	76,6
Jasnic acid2'-Hydroxyflavanone	-9,5	0,3
Naringenin	2,4	-9,4
Quercitrin	-8,9	18,8
Luteolin	-9,4	37,4
Apigenin	-9,4	21,8
Silibinin	-9,3	6,2
EM1404	-12,3	0,0032
EM1424	-10,1	0,0095
EM1396	-11,25	0,013
MPA	-8,8	8,8

BIOINFORMATICS

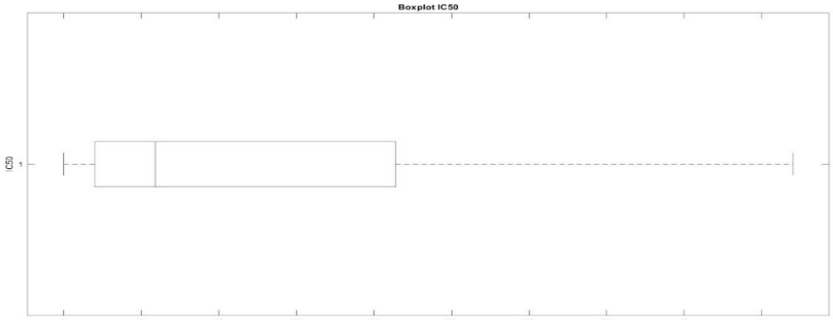

Figure S1 Boxplot outliers

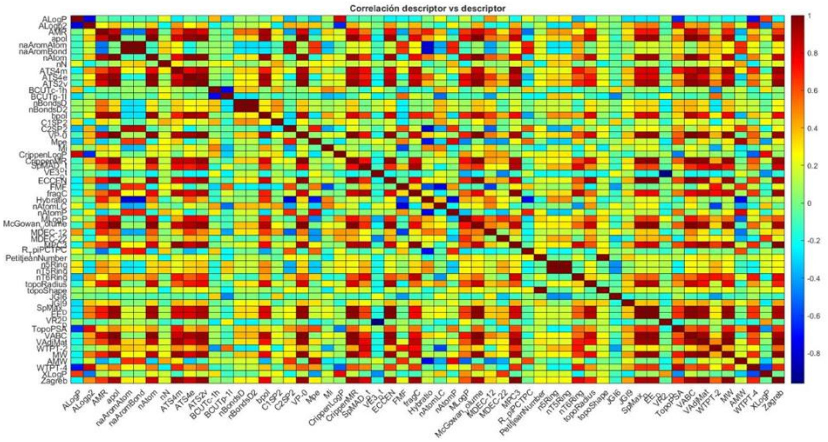

Figure S2 Pearson correlations descriptor vs descriptor

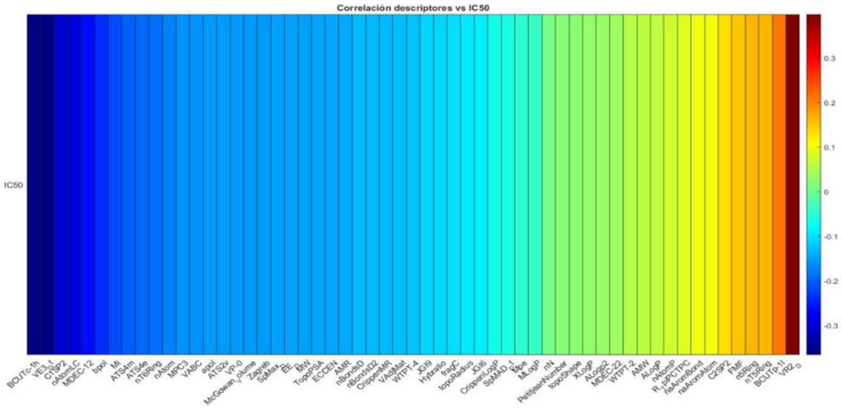

Figure S3 Pearson correlations descriptor vs IC50

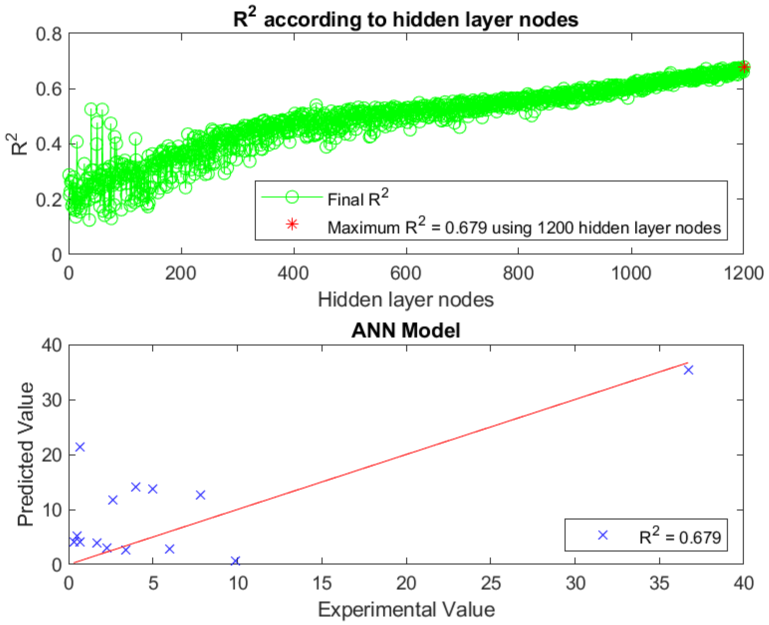

Figure S4 Goodness of fit and cross-validation of the QSAR mode

INTERACTIONS

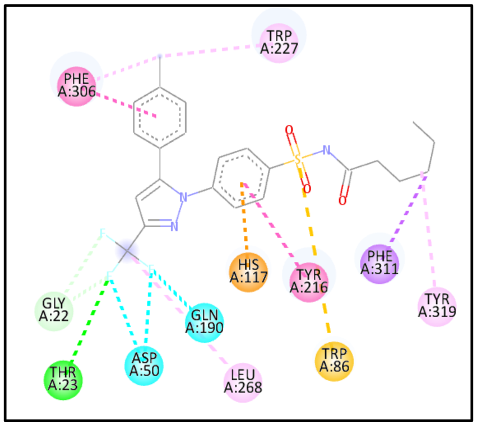

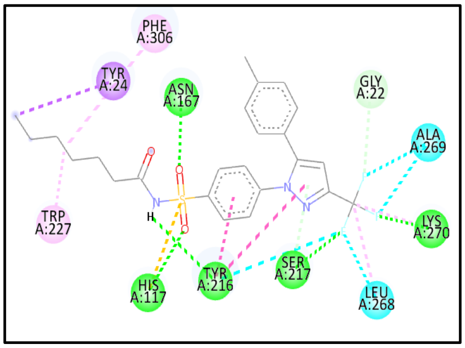

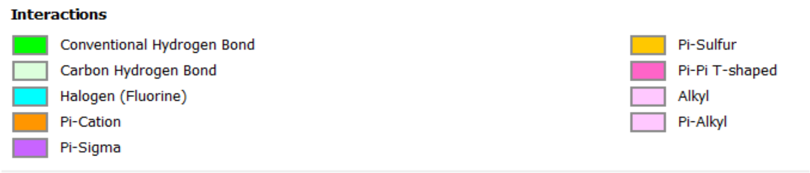

CHEMISTRY

All reagents were used as received from commercial suppliers. Reaction progress was monitored by TLC performed on alumin plates coated with silica gel F_{254} indicator and visualized by either UV irradiation or staining with iodine. Flash Chromatography was carried out by silica gel 60 (230−240 mesh). ^{1}H NMR and ^{13}C NMR spectra were recorded in MEOD using a Bruker Avance NEO 400 MHz spectrometer. Chemical shifts (^{1}H and ^{13}C) are given in parts per million (ppm, δ), from tetramethylsilane as internal reference. ^{1}H NMR splitting patterns were designated as singlet (s), doublet (d), triplet (t), quartet (q) and multiplet (m); Coupling constants are quoted in Hertz (J) and integration. Infrared spectra were recorded using a Bruker Alpha-P ATR FTIR with diamond crystal. High Resolution Mass spectrometry was carried out on an Agilent 5973 (80 eV) spectrometer using electrospray ionization (ESI). All reagents were used as received from commercial suppliers.

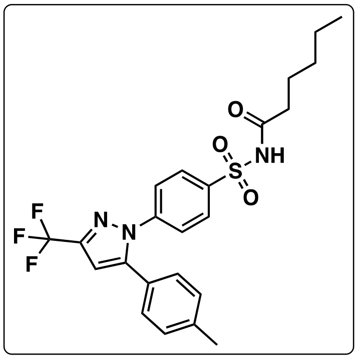

N-((4-(5-(p-tolyl)-3-(trifluoromethyl)-1H-pyrazol-1 yl)phenyl)sulfonyl)hexanamide

^{1}H NMR (400 MHz, Methanol-*d_4*) δ 7.93 (d, *J* = 8.7 Hz, 2H), 7.48 (d, *J* = 8.7 Hz, 2H), 7.09 – 7.30 (m, 4H), 6.90 (s, 1H), 2.34 (s, 3H), 2.27 (t, *J* = 7.5 Hz, 2H), 1.49 – 1.68 (m, 2H), 1.16 – 1.42 (m, 4H), 0.91 (t, *J* = 6.9 Hz, 3H). **^{13}C NMR (101 MHz, MeOD)** δ 177.7, 147.0, 145.0, 144.8 (q, *J* = 38 Hz) 143.2, 141.0, 130.6, 130.0, 128.3, 127.2, 127.0, 122.7 (q, *J* = 268.7 Hz),106.9, 48.6, 34.9, 32.4, 25.8, 23.4, 21.3, 14.3. **FT-IR (neat) u(cm^{-1}):** 3334, 3228, 2928, 1708, 1345, 1133, 1101. **HRMS (ESI):** $C_{23}H_{25}F_3N_3O_3S^+$ [M + H^+]: calc. 480.1563, found. 480.1566.

NMR spectra

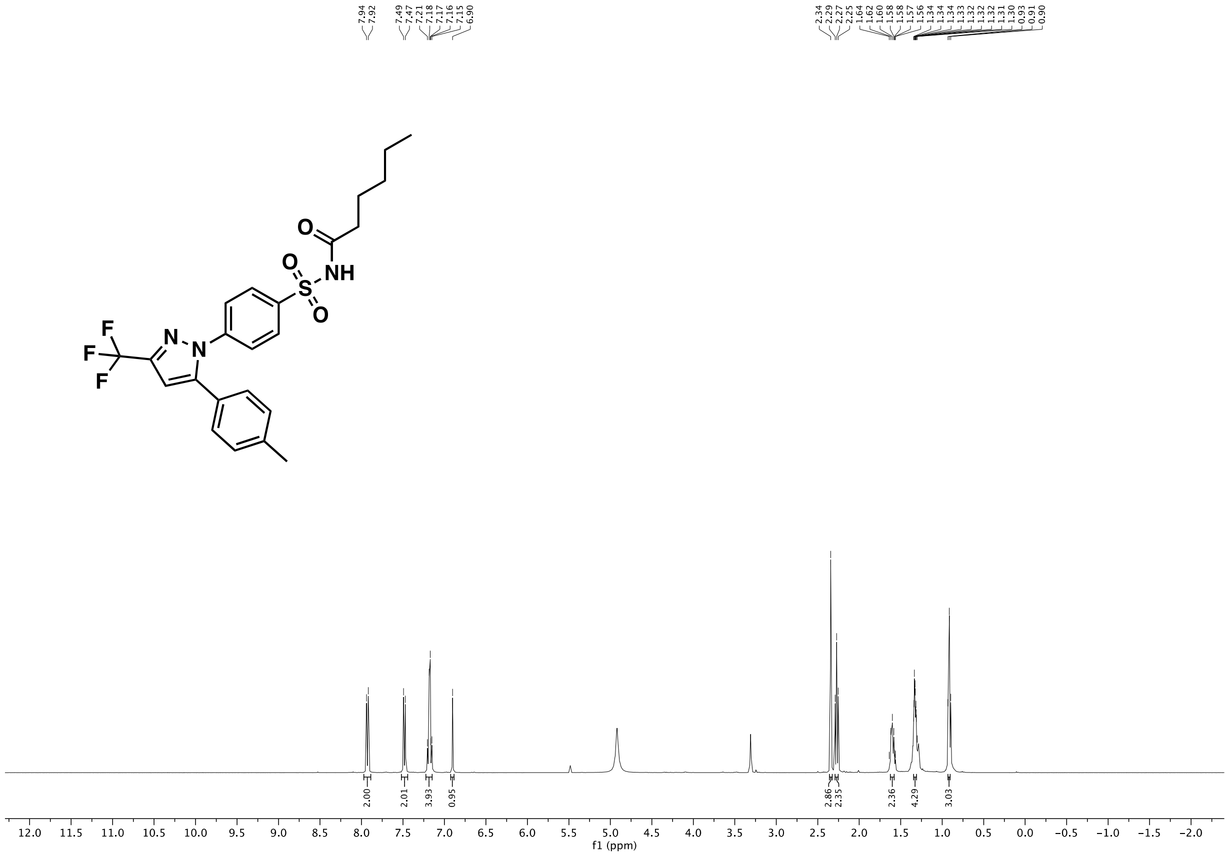
7.94
7.92
7.49
7.47
7.21
7.18
7.17
7.16
7.15
6.90
2.34
2.29
2.27
2.25
1.64
1.62
1.60
1.58
1.58
1.57
1.56
1.34
1.34
1.34
1.33
1.32
1.32
1.32
1.31
1.30
0.93
0.91
0.90
2.00
2.01
3.93
0.95
2.86
2.35
2.36
4.29
3.03
f1 (ppm)

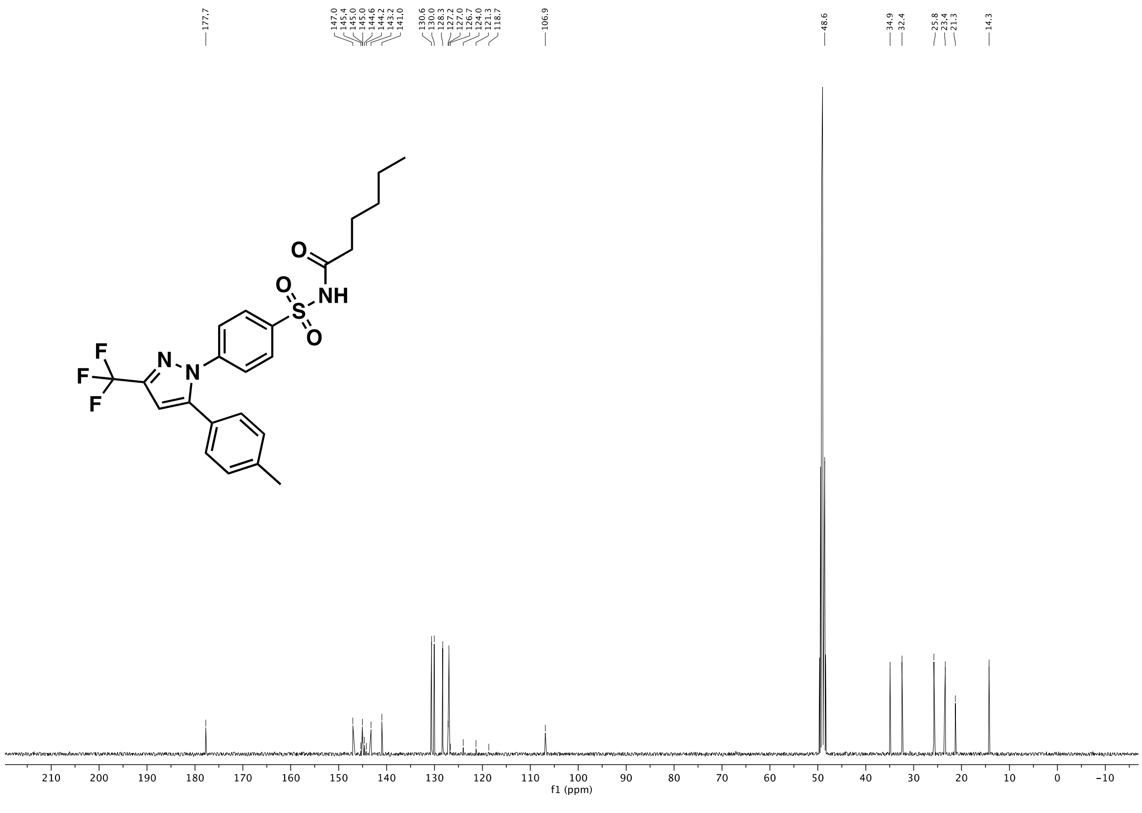
177.7
147.0
145.4
145.0
145.0
144.6
144.2
143.2
141.0
130.6
130.0
128.3
127.2
127.0
126.7
124.0
121.3
118.7
106.9
48.6
34.9
32.4
25.8
23.4
21.3
14.3
f1 (ppm)

IR-ATR Spectra

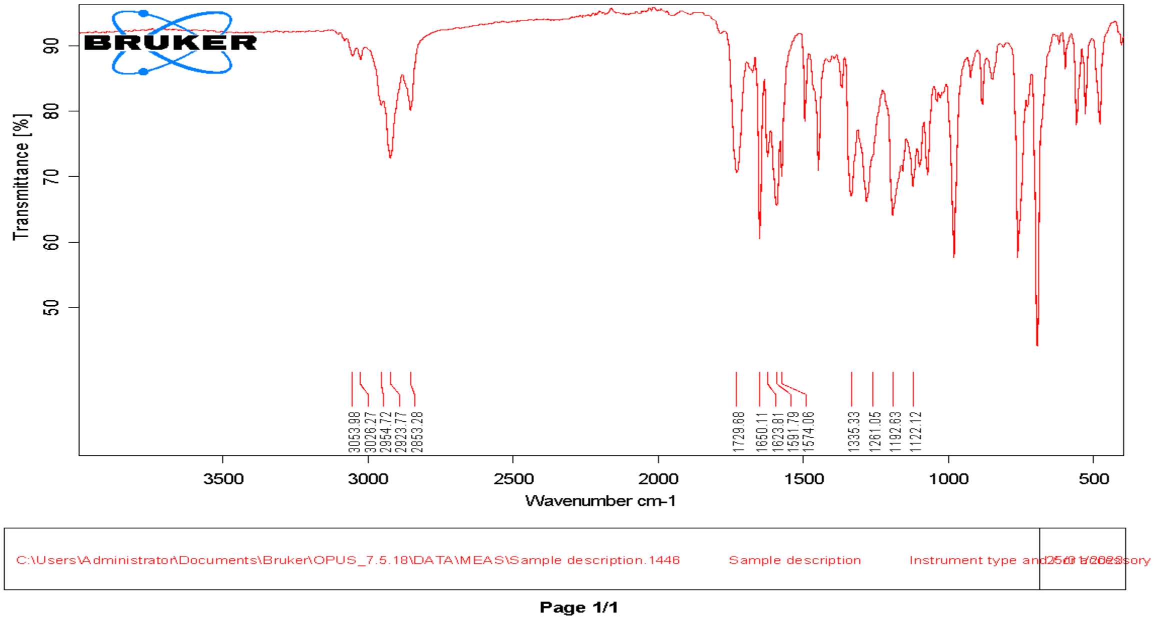

HPLC C-6

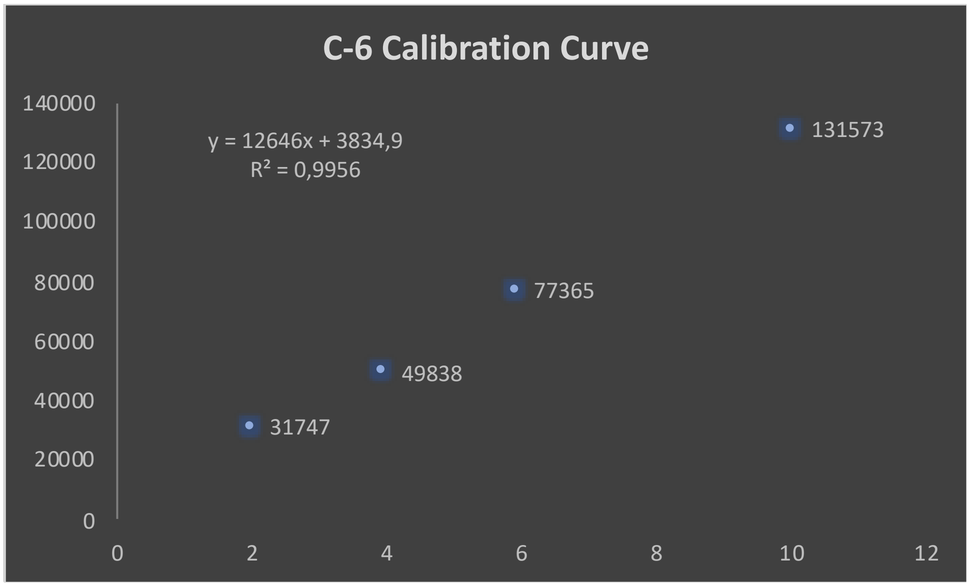

Supplement: Supplementary file 1 [file molecules-29-01802-s001.zip › molecules-2931702-supplementary.pdf]
